# Fatal Necrotizing Fasciitis following Episiotomy

**DOI:** 10.1155/2015/562810

**Published:** 2015-05-07

**Authors:** Faris Almarzouqi, Gerrit Grieb, Christian Klink, Dirk Bauerschlag, Paul C. Fuchs, Ziyad Alharbi, Marketa Vasku, Norbert Pallua

**Affiliations:** ^1^Department of Plastic Surgery, Hand Surgery and Burn Center, Faculty of Medicine, RWTH Aachen University Hospital, Pauwelsstrasse 30, 52074 Aachen, Germany; ^2^Department of General, Visceral and Transplant Surgery, Faculty of Medicine, RWTH Aachen University Hospital, Pauwelsstrasse 30, 52074 Aachen, Germany; ^3^Department of Obstetrics and Gynecology, Faculty of Medicine, RWTH Aachen University Hospital, Pauwelsstrasse 30, 52074 Aachen, Germany; ^4^Department of Plastic and Reconstructive Surgery, Hand Surgery and Burn Care Center, Cologne-Merheim Medical Center, University of Witten/Herdecke, Ostmerheimer Straße 200, 51109 Cologne, Germany

## Abstract

*Introduction*. Necrotizing fasciitis is an uncommon condition in general practice but one that provokes serious morbidity. It is characterized by widespread fascial necrosis with relative sparing of skin and underlying muscle. Herein, we report a fatal case of necrotizing fasciitis in a young healthy woman after episiotomy. *Case Report*. A 17-year-old primigravida underwent a vaginal delivery with mediolateral episiotomy. Necrotizing fasciitis was diagnosed on the 5th postpartum day, when the patient was referred to our tertiary care medical center. Surgical debridement was initiated together with antibiotics and followed by hyperbaric oxygen therapy. The patient died due to septic shock after 16 hours from the referral. *Conclusion*. Delay of diagnosis and consequently the surgical debridement were most likely the reasons for maternal death. In puerperal period, a physician must consider necrotizing fasciitis as a possible diagnosis in any local sings of infection especially when accompanied by fever and/or tenderness. Early diagnosis is the key for low mortality and morbidity.

## 1. Introduction

Necrotizing fasciitis (NF) is an uncommon condition in general practice but one that provokes serious morbidity [1]. It is characterized by widespread fascial necrosis with relative sparing of skin and underlying muscle [2] that can be caused by toxin-producing, aggressive bacteria. Fortunately, the incidence of NF in adults has been reported to be 4 cases per 1 million population [3]. Microbiologically, NF has been classified as either type 1 (polymicrobial) or type 2 (monomicrobial). Polymicrobial infections are more common, with cultures yielding a mixture of aerobic and anaerobic organisms [4]. These infections typically occur in the perineum and trunk. The microbiologic isolates reflect normal skin commensalism found adjacent to the site of infection. For example, in NF of the perineum, anaerobic bacteria are isolated. The etiologic isolates consist of gram-positive organisms, such as* Staphylococcus aureus*,* S. pyogenes*, and enterococci; gram-negative aerobes, such as* Escherichia coli* and* Pseudomonas* species; and anaerobic organisms, such as* Bacteroides* or* Clostridium* species [1, 5, 6]. In the last 3 decades necrotizing fasciitis has been reported in the postpartum period [7, 8]. We report a fatal case of necrotizing fasciitis in a young healthy woman after episiotomy.

## 2. Case Report

**Figure 1:**
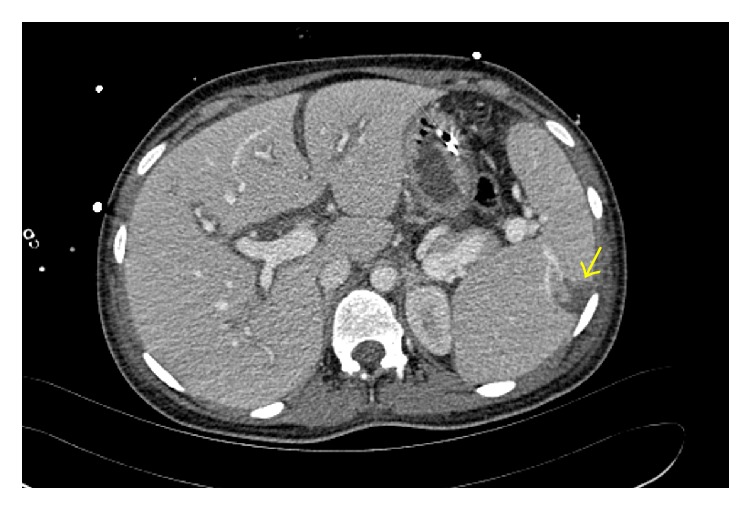


**Figure 2:**
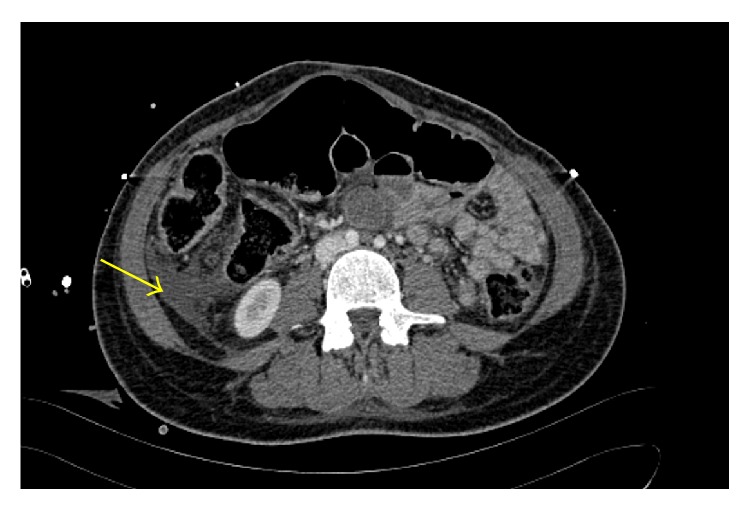


A 17-year-old healthy white Caucasian primigravida underwent a vaginal delivery with mediolateral episiotomy in a secondary care hospital. Her past medical history was unremarkable and the pregnancy was uncomplicated. According to the report of the referring hospital, the patient complained of local pain at that time, which was considered as normal after birth. The white blood cells were high in the first postpartum day (23.81/nL) and were decreasing in the second (13.69/nL), third (8.42/nL), and fifth (2.98/nL) postpartum day showing a beginning of leukocytopenia. In the fourth postpartum day, the patient developed a severe pain and edema in the episiotomy site as well as the lower extremities. Further on, the patient exhibited a progressive purpuric discoloration on the right leg radiating from the episiotomy site, which was accompanied by general deterioration. In the early hours of the fifth postpartum day, the patient was admitted to the intensive care unit (ICU) under antibiotic therapy (Piperacillin/Combactam, Metronidazole, and Clindamycin). Ultrasound showed perifascial fluids in the suprapubic region and in both groins to the iliac vessels, which extends downwards to the medial aspect of both thighs. Chest X-ray demonstrated a cardiomegaly with pulmonary congestion with no evidence of pleural effusion. Duplex ultrasound ruled out thrombophlebitis of both lower extremities. In the late morning hours of that day, the patient was referred to our tertiary care medical center. Due to the instable status, the patient had to be intubated and under high dose of catecholamine. Vital signs at the time of admission were as follows: blood pressure 140/90 mmHg, respiratory rate 20/min, pulse 90/min, and temperature 35.5°C. Both upper extremities presented with massive swelling and signs of compartment syndrome. Both lower extremities were massively swollen with discoloration (purple to red) of the skin more on the right side. Computer tomography (CT) scan was performed to the trunk and extremities. The scan showed bilateral pleural effusion and atelectasis, splenomegaly and partial splenic infarction (2 cm) (Figure 1), free intra-abdominal fluid (Figure 2), anasarca, and edematous lower limbs especially the muscles (right > left) as well as thrombosis of the great saphenous vein on the right side. After the emergent initial investigations, NF was diagnosed clinically and the antibiotic therapy was adjusted to Clindamycin and Penicillin G. The patient was driven to the operating theater with low lactic acid and pH in the arterial blood gasses with value of 3.7 mg/dL and 7.26, respectively. These values have been normalized in the following 2 hours. An interdisciplinary team from the departments of general surgery, plastic surgery, and gynecological surgery initiated a radical debridement of the necrotic tissues of the perineum and pelvic floor as well as fasciotomy of the four extremities. Multiple biopsies of the fascia were sent to the department of pathology as well as department of microbiology for immediate examination. All cultures of the samples showed* Streptococcus pyogenes*. Some of the samples showed* Bacteroides fragilis* and* Escherichia coli* as well. Interestingly, the patient remained with good renal and liver functions together with adequate urine output. Thus, a suspicion of an increase in intra-abdominal pressure as well as abdominal compartment syndrome was ruled out and a decompressive laparotomy was not indicated. However, the myoglobin in urine exceeded the maximum measurable value in our laboratory (>3000 *μ*g/L). Intraoperatively the patient developed light fixed pupils. An emergent cranial CT, after the initial emergent surgery, ruled out any hypoxia or intracranial bleeding. The blood work-up test at this time revealed signs of disseminated intravascular coagulopathy (DIC): platelet count = 10 g/L, TPZ (Quick) = 43%, partial thromboplastin time (PTT) = 57 sec., fibrinogen = 1.2 *μ*g/L, and D-dimers = 6778 *μ*g/L. Therefore, the patient required sixteen units of packed red blood cells (250 mL each), twelve units of fresh frozen plasma, and four units of concentrated platelets (250 mL each) while being in our hospital. After the surgery and as a supportive therapy, the patient was referred to hyperbaric oxygen therapy (HBO). The vital signs were at that time as follows: blood pressure 110/70 mmHg, respiratory rate 14/min, pulse 100/min, and temperature 37°C under high dose of catecholamine. After three hours in the HBO chamber the patient dropped with blood pressure and consequently catecholamine therapy was escalated. After being transferred back to our ICU, a transthoracic echocardiogram has been carried out and showed massively dilated ventricles on both sides and reduced cardiac output with global cardiac hypo- to akinesia. Short after, the patient had to be resuscitated according to European Resuscitation Council (ERC). Resuscitation attempts failed and the patient died due to septic shock after 16 hours since hospital transfer.

## 3. Discussion

Necrotizing fasciitis was actually first described by Hippocrates in the 5th century B.C. He discussed it as a complication of “erysipelas” [9]. Furthermore, he described it as the most dangerous NF of all kinds when it comes to the genital area: “In some cases the entire thigh was bared or the shin and the entire foot. But the most dangerous cases of all such cases were when the pubes and genital organs were attacked” [9]. In the last century, the reported mortality rate was between 6% and 76% [10].

NF occurs more frequently in patients suffering from diabetes, alcohol addiction, immunosuppressive diseases, and IV drug addiction and patients with peripheral vascular diseases [2]. However, we could not identify any of these risk factors in our patient. In addition to previous risk factors and based on gynecological point of view, endomyometritis, parametritis, and adnexitis are known to be differential diagnosis of puerperal fevers. Episiotomies could be sometimes very painful and accompanied by drop of WBCs count instead of increasing on the early postpartum days; therefore, a high suspicion of serious conditions may avoid the patient from fatal complications like necrotizing fasciitis that can be misdiagnosed or underestimated easily. NF can be mostly diagnosed clinically in its early phase. Typically, patients with a predisposing factor and history of minimal trauma begin to have unexplained severe and continuous pain and go through rapid deterioration in general health “feel worse than they have ever felt and don't know why” [11]. Thus, close observation in these clinical scenarios including regular measurements of vital signs, fluid balance assessment, blood work-up tests and cultures, wound monitoring, and swab cultures is necessary. Imaging studies like CT scan and MRI can be helpful suggesting early diagnosis but cannot rule out NF [12]. On the other hand, sepsis in postpartum period remains an important cause of maternal death [13]. Risk factors for maternal sepsis are obesity, diabetes, immunosuppression, anemia, vaginal discharge, cervical cerclage, vaginal trauma, history of pelvic infection, and prolonged rupture of membrane [14]. Physicians should be aware of signs and symptoms (Table 1) [14] of maternal sepsis and critical illness and of the rapid, potentially lethal course of severe sepsis and septic shock. Suspicion of significant sepsis should trigger urgent referral to secondary or tertiary care.

McHenry et al. found that delays in diagnosis and treatment of NF (particularly adequate surgical debridement and fasciotomy) correlated with poor outcome [10]. In our case despite the aggressive surgical debridement supported by hyperbaric oxygen therapy, which is able to lower the mortality rate to 12% [15], the patient died likely due to the massive extension of the disease and the delay of diagnosis.

## 4. Conclusion

In puerperal period, a physician must consider the NF as a possible diagnosis in any local sings of infection especially when accompanied by fever and/or local tenderness. Early diagnosis and transfer of the patient to specialized hospital are the keys for low mortality and morbidity in this life threatening disease.

## Figures and Tables

**Table 1:** Common signs and symptoms in puerperal sepsis.

Signs	Symptoms
(i) Fever (ii) Wound infection (iii) Breast engorgement/redness (iv) Rash (v) Delay in uterine involution (vi) Offensive vaginal discharge	(i) Rigors (ii) Diarrhea or vomiting (iii) Productive cough (iv) Urinary symptoms (v) Abdominal/pelvic pain (vi) Fatigue and reduced appetite
